# Kinetic modelling and quantification bias in small animal PET studies with [^18^F]AB5186, a novel 18 kDa translocator protein radiotracer

**DOI:** 10.1371/journal.pone.0217515

**Published:** 2019-05-31

**Authors:** Mark G. MacAskill, Tashfeen Walton, Lewis Williams, Timaeus E. F. Morgan, Carlos José Alcaide-Corral, Marc R. Dweck, Gillian A. Gray, David E. Newby, Christophe Lucatelli, Andrew Sutherland, Sally L. Pimlott, Adriana A. S. Tavares

**Affiliations:** 1 University/ BHF Centre for Cardiovascular Science, University of Edinburgh, Edinburgh, United Kingdom; 2 Edinburgh Imaging, University of Edinburgh, Edinburgh, United Kingdom; 3 WestCHEM, School of Chemistry, University of Glasgow, Glasgow, United Kingdom; 4 School of Medicine, University of Glasgow, Glasgow, United Kingdom; 5 West of Scotland PET Centre, NHS Greater Glasgow and Clyde, Glasgow, United Kingdom; Biomedical Research Foundation, UNITED STATES

## Abstract

**Introduction:**

Positron Emission Tomography (PET) imaging with selective 18 kDa translocator protein (TSPO) radiotracers has contributed to our understanding on the role of inflammation in disease development and progression. With an increasing number of rodent models of human disease and expansion of the preclinical PET imaging base worldwide, accurate quantification of longitudinal rodent TSPO PET datasets is necessary. This is particularly relevant as TSPO PET quantification relies on invasive blood sampling due to lack of a suitable tissue reference region. Here we investigate the kinetics and quantification bias of a novel TSPO radiotracer [^18^F]AB5186 in rats using automatic, manual and image derived input functions.

**Methods:**

[^18^F]AB5186 was administered intravenously and dynamic PET imaging was acquired over 2 hours. Arterial blood was collected manually to derive a population based input function or using an automatic blood sampler to derive a plasma input function. Manually sampled blood was also used to analyze the [^18^F]AB5186 radiometabolite profile in plasma and applied to all groups as a population based dataset. Kinetic models were used to estimate distribution volumes (*V*_*T*_) and [^18^F]AB5186 outcome measure bias was determined.

**Results:**

[^18^F]AB5186 distribution in rats was consistent with TSPO expression and at 2 h post-injection 50% of parent compound was still present in plasma. Population based manual sampling methods and image derived input function (IDIF) underestimated *V*_*T*_ by ~50% and 88% compared with automatic blood sampling, respectively. The *V*_*T*_ variability was lower when using IDIF versus arterial blood sampling methods and analysis of the Bland-Altman plots showed a good agreement between methods of analysis.

**Conclusion:**

Quantification of TSPO PET rodent data using image-derived methods, which are more amenable for longitudinal scanning of small animals, yields outcome measures with reduced variability and good agreement, albeit biased, compared with invasive blood sampling methods.

## Introduction

The 18 kDa translocator protein (TSPO), previously known as the peripheral benzodiazepine receptor, is a five-membrane domain protein expressed in the outer membrane of the mitochondria of different cell types present in peripheral tissues and the central nervous system (CNS). In the periphery, high TSPO levels are reported in immune cells, including macrophages [1–3]. In the CNS, high levels of TSPO are associated with activated microglia and astrocytes [4–8]. At the organ level, the highest expression of TSPO is reported in the lungs and in the heart, and low levels are present in the healthy brain [9,10].

Given the expression of TSPO in immune cells, TSPO imaging is an attractive target for Positron Emission Tomography (PET) imaging of inflammation. To date, TSPO PET imaging has been used to non-invasively investigate regional inflammatory responses in a wide array of human diseases, including Parkinson’s disease [11], Alzheimer’s disease [12], traumatic brain injury [13], stroke[14], myocardial infarction [15] and atherosclerosis [1,2]. Moreover, over the past few years, translational TSPO small animal PET imaging has rapidly expanded due to increasing availability of rodent models of human disease and preclinical PET scanners. TSPO radiotracers have progressively been used to interrogate disease development and progression, as well as treatment response in rodents.

Despite the translational value of TSPO PET imaging in multiple diseases, TSPO radiotracers developed to date have suboptimal properties, hampering their widespread clinical use. ^11^C-PK11195 was the first radiotracer to be used consistently for *in vivo* imaging of inflammation using PET, but has high levels of non-specific binding[16], high plasma protein binding[17] and a short physical half-life due to carbon-11 (20 minutes). Second generation TSPO ligands (e.g. [^11^C]PBR028 and [^18^F]PBR111) display large interindividual variability in binding affinity in humans, which has been attributed to a human genetic polymorphism [18]. The limitations of previously developed TSPO radiotracers have led to multiple research groups, including ourselves, to work on developing new libraries of novel compounds targeting TSPO.

In addition to difficulties in generating a TSPO radiotracer with optimal properties for *in vivo* imaging of humans, TSPO PET imaging is also devoid of a good tissue reference region for simplified quantification of data. As such, absolute quantification relies typically on the use of invasive blood input functions. This characteristic can pose limitations to clinical imaging with TSPO radiotracers, and the problem is further complicated when imaging small animals, owing to the small size and limited blood volume of rodents.

Therefore, studies investigating the quantitative bias of different TSPO PET data analysis approaches (invasive versus non-invasive) in small animals would inform on optimal protocols to better evaluate changes in TSPO expression in rodent models of disease, as well as measurement of new radiotracers and drugs performance with TSPO PET imaging. Recently we developed [^18^F]AB5186, a promising novel TSPO radiotracer, and assessed its *in vivo* performance in a rodent model of glioblastoma and in the brain of a baboon [17]. This radiotracer displayed good brain imaging properties similar to other TSPO PET radiotracers currently in preclinical and clinical use. The present study aimed to assess the quantification bias in small animal PET kinetic modelling studies using [^18^F]AB5186 and invasive versus non-invasive quantification methods, and specifically to compare the most widely reported methods for generation of an input function in rodent PET studies (image derived and population curves) with invasive and continuous automatic blood sampling.

## Materials and methods

### Radiotracer preparation

**Fig 1:**
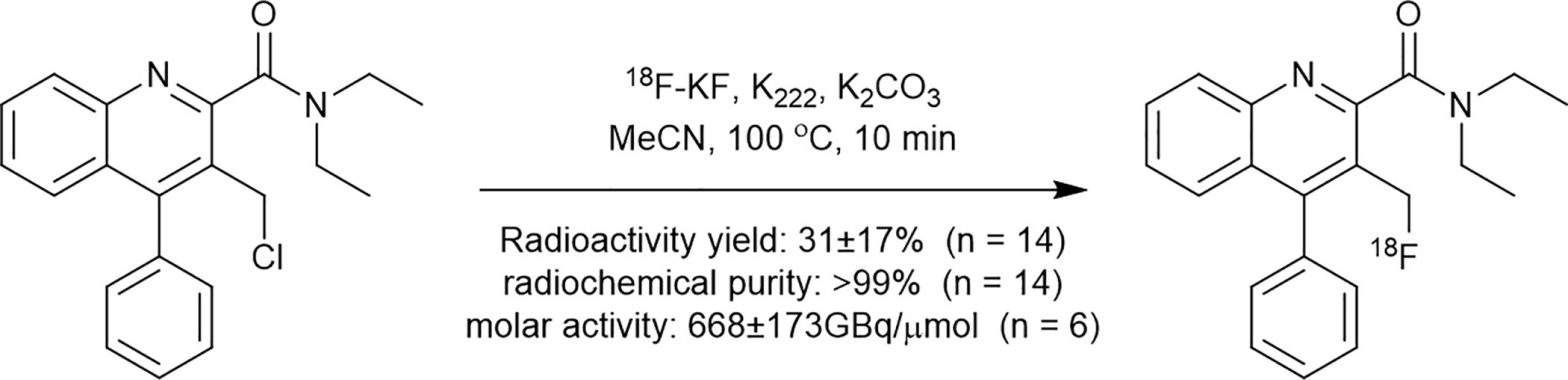
[^18^F]AB5186 radiosynthesis.

AB5186 precursor and standard were prepared from 2-aminobenzophenone and diethyl acetylenedicarboxylate in five and six steps, respectively, as described in S1 Data. [^18^F]AB5186 was prepared by reacting 3-chloromethyl-4-phenylquinoline-2-*N*-diethylcarboxamide precursor with ^18^F-fluoride in the presence of potassium carbonate and Kryptofix 222 at 100°C for 10 min (Fig 1), using a commercial synthesizer, GE TRACERlab FX-_FN_. The radiolabeled product was purified by semi-preparative High-Performance Liquid Chromatography (HPLC) using the following conditions: C18 Synergi Hydro-RP 80Å, 150×10mm, 4μm column (Phenomenex, UK), acetonitrile/water (70:30 v/v) and flow rate of 3 mL/min. The final product was formulated in a physiological solution containing 10% ethanol in normal saline. [^18^F]AB5186 was obtained with an average activity yield of 31% (starting from 15±6 GBq of ^18^F-fluoride, n = 14) after a total synthesis time of 50 minutes. Identity of [^18^F]AB5186, radiochemical purity (>99%) and specific activity (495–900 GBq/μmol; 13–24 Ci/μmol, n = 6) were determined by analytical HLPC at the end of the synthesis using the following conditions: C18 Synergi Hydro-RP 80 Å, 250×4.6 mm, 4μm column (Phenomenex, UK), acetonitrile/water (70:30 v/v), flow rate of 1 mL/min and ultraviolet absorbance at λ = 267 nm.

### Animals and surgical procedures

All experiments were conducted in accordance with the local University of Edinburgh animal ethics committee and were authorized by the Home Office under the Animals (Scientific Procedures) Act 1986. Thirteen adult male Sprague-Dawley rats (416±79g and 11.0±0.3 weeks) were used for this study. The animals were housed under standard 12 h light:12 h dark conditions with food and water available *ad libitum*. On the day of the experiment, anesthesia was induced and maintained with 1.5–2.5% isoflurane (50/50 oxygen/nitrous oxide, 1 L/min). At the end of experiments animals where euthanized by overdose of anesthesia followed by confirmation of death using a schedule 1 method (cervical dislocation). For imaging experiments, an intravenous (i.v.) line was established in the femoral vein or tail vein for injection of the radiotracer and the femoral artery was cannulated to allow automated blood sample collection, as previously described [19]. On a separate set of experiments (radiometabolite studies), the femoral artery was cannulated for blood sampling and the radiotracer was administered i.v. via tail vein. Surgical cannulation of femoral vein and artery was performed as follows: polyethylene catheters (PE50) filled with heparinized saline (20 IU/mL) were inserted into the left femoral artery or vein with the help of a stereomicroscope and securely fastened with ligatures (6–0 silk thread). Catheters were held in place with surgical glue. Body temperature was maintained by heated scanner bed or heated mat and monitored by rectal thermometer. Vital signs, including heart rate and respiration rate were monitored continuously during the experiments.

### PET studies

#### Study design

PET scans were performed immediately following intravenous bolus injection of [^18^F]AB5186 (mean injected radioactivity of 27.87±2.51 MBq, mean±SD, n = 3) via the femoral vein shunt set-up for automatic blood sampling, as previously described [19]. PET dynamic imaging data collected as part of these kinetic modelling experiments were modelled using three different input function methods: (Group 1) automatic blood sampling with the Swisstrace system for each individual animal scanned (gold-standard method), (Group 2) manual blood sampling through the femoral artery to create a population curve from various animals as well as to derive a radiometabolite population curve data (sampling details below), and (Group 3) image derived input function from the left ventricle VOI for each individual animal scanned. All input functions were modelled using the population radiometabolite curve.

#### Image acquisition and reconstruction

All PET data were acquired using a microPET/CT animal scanner (nanoPET/CT, Mediso, Hungary). A CT scan (semi-circular full trajectory, maximum field of view, 480 projections, 50 kVp, 300 ms and 1:4 binning) was acquired for attenuation correction. Immediately following radiotracer administration, a 120 minutes emission scan was obtained using 3-dimentional 1:5 mode and re-binned as follows: 18×10sec; 2×30sec; 1×60sec; 2×2min; 10×5min; 6×10min. PET images were reconstructed using Mediso’s iterative Tera-Tomo OSEM 3D reconstruction algorithm and the following settings: 4 iterations, 6 subsets, full detector model, low regularization, spike filter on, voxel size 0.4 mm and 400–600 keV energy window. PET data were corrected for randoms, scatter and attenuation.

#### Image processing

Reconstructed scans were imported into PMOD 3.8 software (PMOD Technologies, Switzerland). Volumes of interest (VOIs) were manually drawn around lungs, heart and brain; and a 1 mm radius sphere (equivalent to 4.18 mm^3^ and within acceptable range given the PET scanner resolution of 0.7 mm) was used as blood pool and placed inside the left ventricle. The VOI rat brain template (W. Schiffer) [20] available on PMOD was used for PET image co-registration and VOI placement for quantification of regional brain radiotracer uptake kinetics. Average images of [^18^F]AB5186 PET data were generated by averaging frames with highest brain uptake (0–30 min post-radiotracer administration for all subjects). Then, the average image was co-registered to the rat MRI template available in PMOD using rigid manual matching. The transformation matrix was saved and subsequently applied to the dynamic PET series. Finally, template VOIs were applied to the co-registered PET images for image quantification. The following template brain regions were analyzed: striatum, frontal association cortex, medial prefrontal cortex, orbitofrontal cortex, enthorhirnal cortex, hypothalamus, thalamus, midbrain, cerebellum white matter and cerebellum grey matter.

#### Data analysis

Time-activity curves (TACs) were generated and standardized uptake values (SUVs) calculated as concentration in the VOI divided by injected dose divided by animal weight.

Kinetic modelling was performed using 2-Tissue compartmental analysis (2T model) and Logan graphical analysis to estimate the volume of distribution (*V*_*T*_) in different brain regions, as well as heart and lungs [21,22]. For kinetic modelling analysis using input function derived from population curves (manual sampling methods), the whole blood and plasma values per subject were all corrected for differences in injected dose, in order to derive meaningful outcome measures. The Akaike selection criteria (AIC) for different models was outputted and the selected kinetic model identifiability criterion was the percentage standard error (%SE) of *V*_*T*_ estimates. The typical error of measurement (TEM) for each method of analysis was calculated as TEM = SD/SQR(2) and the coefficients of variation (COV) were estimated as COV = (SD/Mean)*100. Bland-Altman plots were calculated as previously described [23,24].

### Arterial input functions using the automatic blood sampler

A commercially available automatic blood sampling system (Twilite2, Swisstrace, Switzerland) was used for the measurement of blood radioactivity (Group 1) as previously described [19]. This system allows for whole-blood arterial input function measurements with a temporal resolution of 1 second and without blood loss due to surgically induced arteriovenous shunt. With data acquired in separate studies, the whole-blood arterial input function measured by the automatic blood sampler was corrected for the plasma-to-whole blood ratio and for metabolism *in vivo* (details in section below).

### Radiometabolite and arterial blood processing and analysis

Arterial blood samples were collected at 2, 5, 10, 20, 30, 60 and 120 minutes post-radiotracer administration (65.28±28.37 MBq, mean±SD, n = 10). Brain and heart samples were collected at 120 min post-injection. All blood samples were 1–2 mL each and manually collected from different animals to generate a population curve (n = 3 for each time point), in order to respect total blood volume limits for terminal arterial blood collections in rats. Following blood and tissue collection, all samples were kept on ice until analyzed. Radioactivity in whole blood and plasma was assessed using a well-type γ-counter using a 400–1400 keV window (Perkin Elmer Wizzard2, USA) and used as the input function for Group 2. Brain and heart tissue samples were homogenized, then radioactivity measured in the well-type γ-counter. Plasma samples (400 μL) and tissue samples (2 mL) were processed by acetonitrile denaturation (ratio of 1:1.4) and analyzed by HPLC (Untimate2000, ThermoFisher, UK) on a Luna C18(2) column (Luna C18(2), 10×250 mm, 10 μm, Phenomenex, UK) with a mobile phase of acetonitrile/water 70/30 at a flow rate of 4 mL/min to estimate the parent fraction. The plasma protein binding free fraction (*f*_*p*_) was determined using ultrafiltration units (Centrifree 30K, Millipore, UK).

## Results

**Fig 2:**
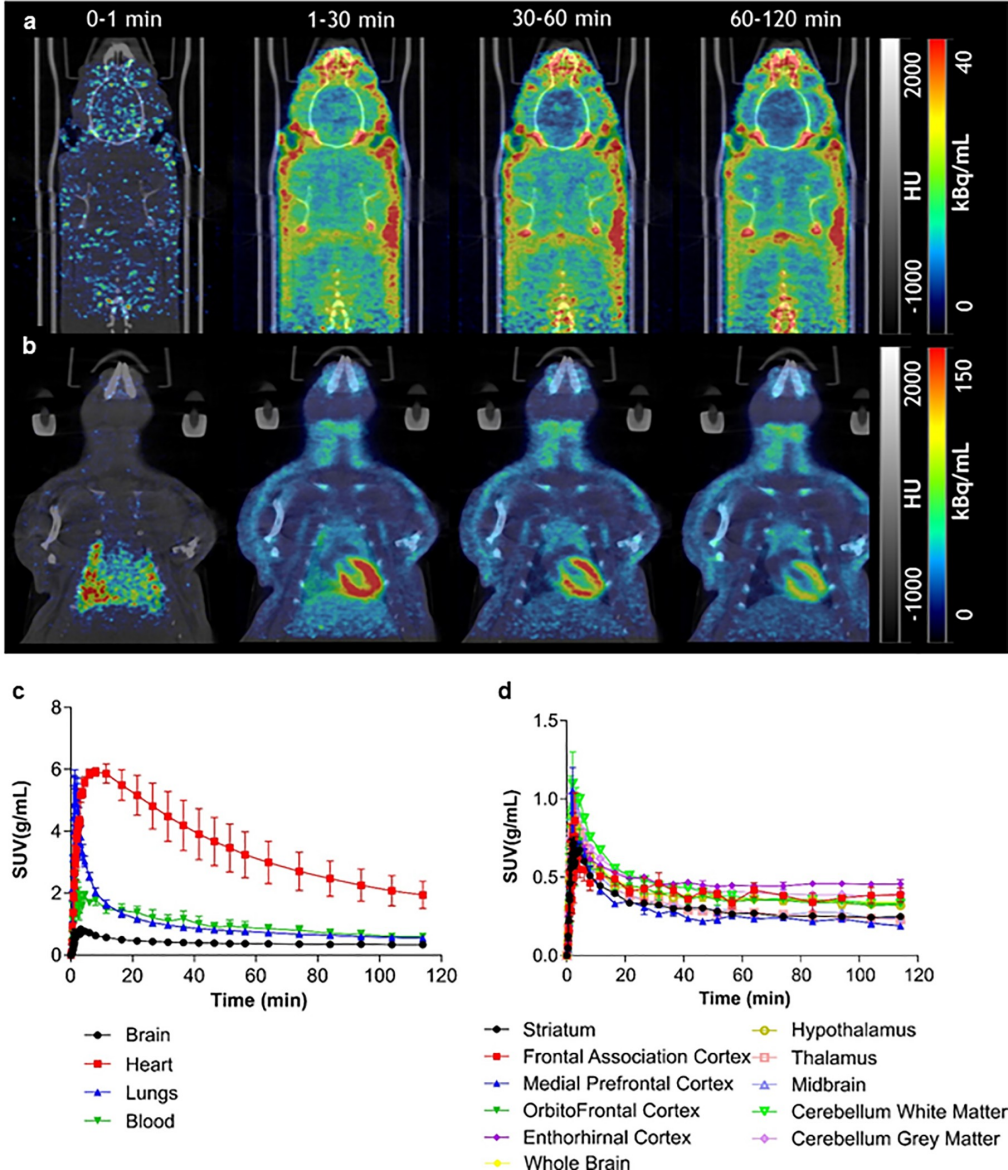
Distribution and kinetics of [^18^F]AB5186 in rats. **(a)** Representative PET sum images of [^18^F]AB5186 at different times points showing distribution of novel radiotracer in rat brain and **(b)** heart and lungs. PET images co-registered with CT images. Average SUV TACs in brain, heart, lungs and blood pool **(c)**, as well as, different brain regions **(d)** following bolus intravenous injection of [^18^F]AB5186. Note reversible tissue kinetics of novel TSPO PET radiotracer. Data presented as mean±SEM, n = 3.

PET studies in rats showed that, following intravenous (i.v.) bolus injection, [^18^F]AB5186 rapidly entered the brain with peak SUV in whole brain of 0.83±0.05 g/mL (mean±SD, n = 3) and displayed a distribution consistent with known TSPO expression, being highest in the heart and lungs (5.92±0.24 g/mL and 4.87±1.93 g/mL, respectively, mean±SD, n = 3) and lowest in the brain (Fig 2). The radiotracer uptake profile was consistent with reversible binding kinetics. Regional TACs in whole organs and brain regions following i.v. bolus of [^18^F]AB5186 are shown in Fig 2C and 2D, respectively.

**Fig 3:**
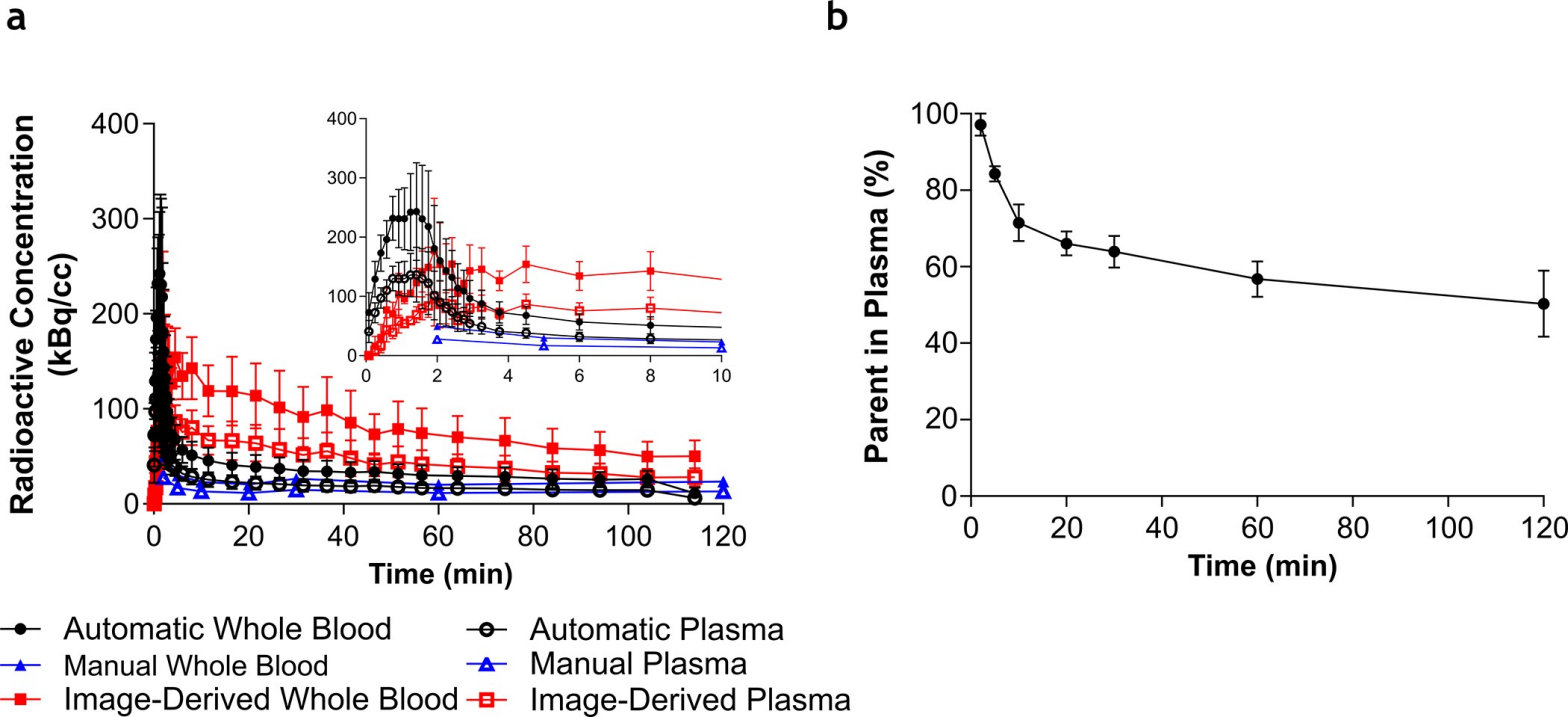
Concentration of [^18^F]AB5186 and parent fraction in rat whole blood and plasma. Normalised measured radioactive concentration in whole blood and plasma using different sampling methods **(a)** and parent fraction profile in plasma over time following bolus intravenous injection of [^18^F]AB5186. The insert graph shows the same data between 0–10 minutes. **(b)**. Automatic whole blood and plasma refer to measurements using the automatic blood sampler system. Manual whole blood and plasma refer to measurements using manual sampling of arterial blood. All data has been normalized relative to injected dose, so that the different whole blood and plasma curves can be compared with each other. Data presented as mean±SEM, n = 3.

Post-i.v. bolus injection, a rapid clearance of [^18^F]AB5186 in rat blood was measured by automatic blood sampling and manual population based time-point sampling (Fig 3A). The image derived input function (VOI in left ventricle) showed similar peak uptake kinetics, but slower plateau kinetics, compared with invasive methods of arterial input function sampling. Metabolism of the novel radiotracer measured by high performance liquid chromatography (HPLC) showed a relatively slow in *vivo* metabolism of [^18^F]AB5186 in blood (Fig 3B) and at 2 h post-injection, 50.31±14.92% (mean±SD, n = 3) of parent was detected in plasma (S1 Fig). No radiometabolites were detected in brain and heart tissue samples 2 h post-injection (S1 Fig). The measured free fraction in plasma (*f*_*p*_) of [^18^F]AB5186 was 6.73±0.01% (mean±SD, n = 3).

**Fig 4:**
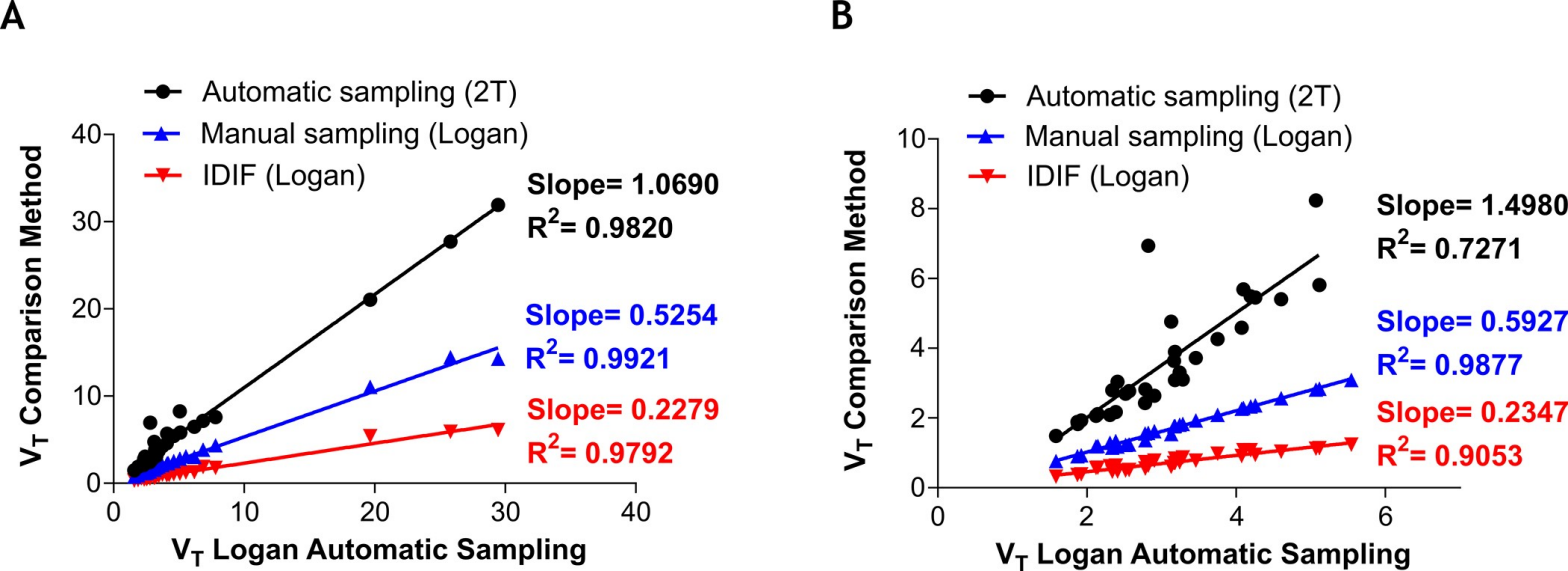
**Comparative analysis between *V***_***T***_ **obtained using 2T model and Logan graphical analysis with different input functions for all tissues (a) and brain regions only (b).** Note strong correlation despite bias of manual sampling and image derived input functions relative to automatic blood sampler. IDIF, image derived input function.

**Fig 5:**
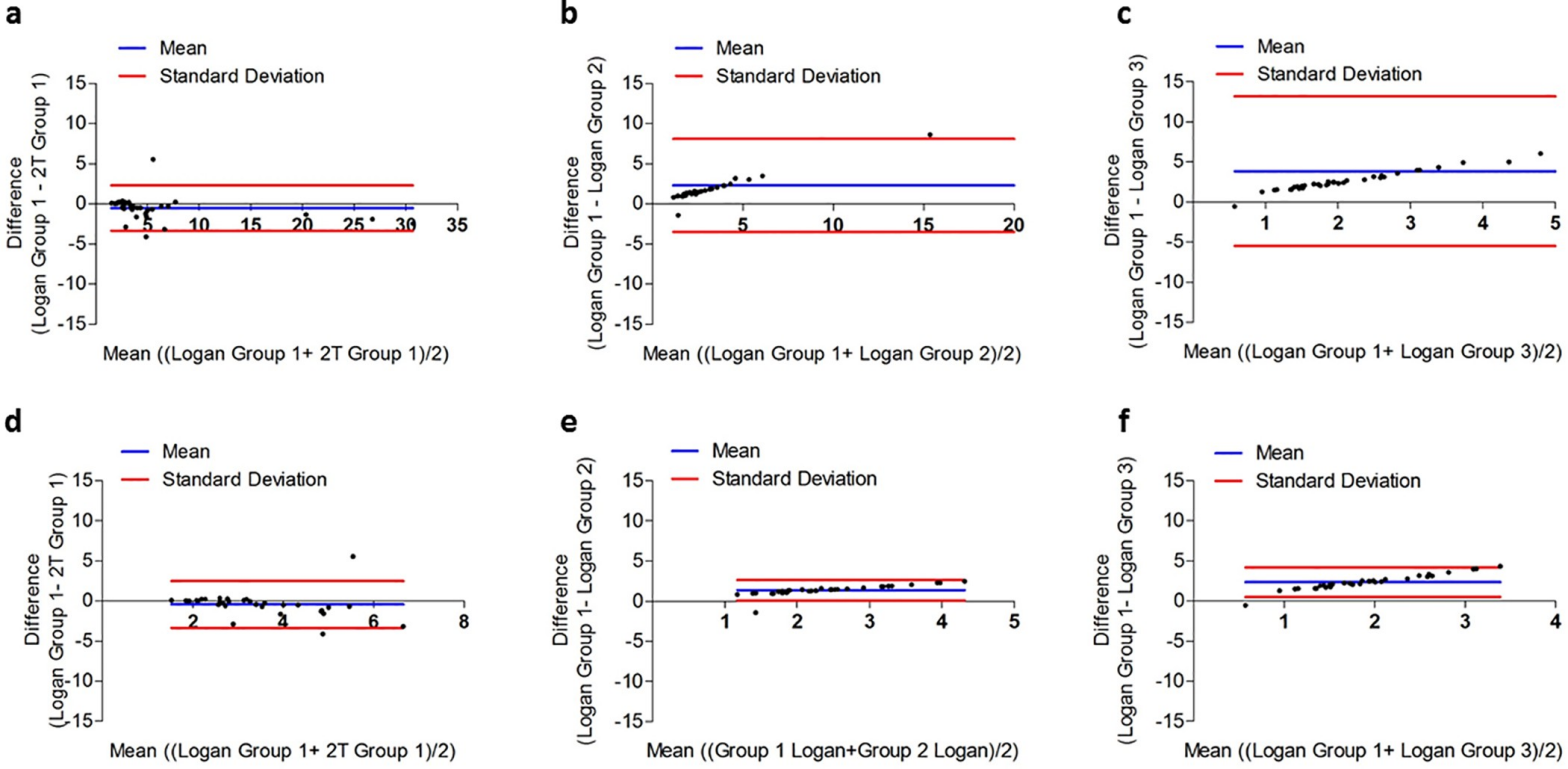
Bland-Altman plots assessing agreement between 2T model and Logan graphical analysis in all tissues with different input functions methods. Group 1 (Logan) and Group 1 (2T) **(a)**; Group 1 (Logan) and Group 2 (Logan) **(b)**; and Group 1 (Logan) and Group 3 (Logan) **(c)**. This was also carried out using only brain regions for Group 1 (Logan) and Group 1 (2T) **(d)**; Group 1 (Logan) and Group 2 (Logan) **(e)**; and Group 1 (Logan) and Group 3 (Logan) **(f)**.

**Fig 6:**
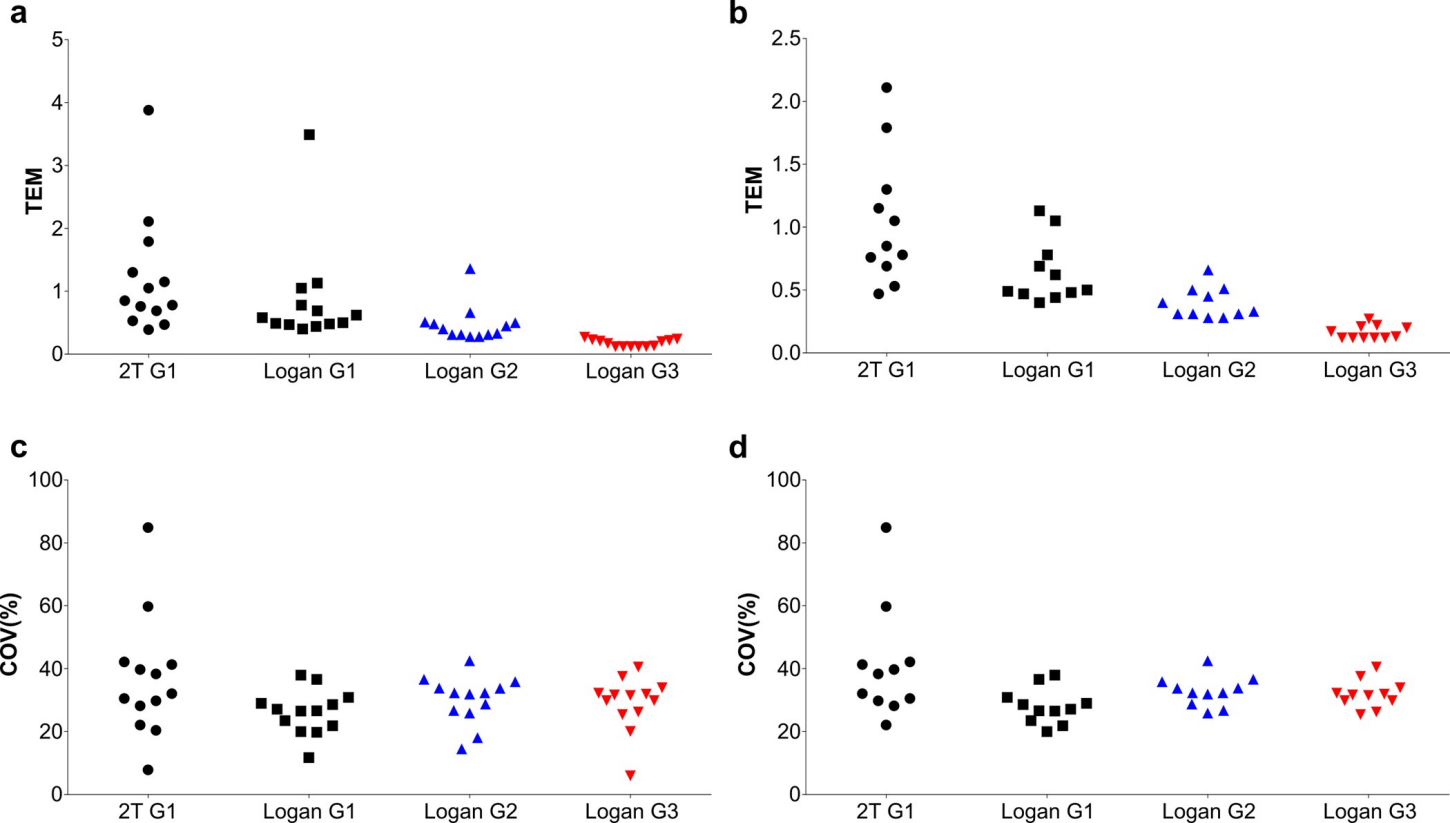
Calculated typical error of measurement (TEM) and coefficients of variation (COV). **(a)** TEM of tissues and **(b)** brain regions for all methods investigated. **(c)** %COV of tissues and **(d)** brain regions for all methods investigated. G1 = group 1 (automatic blood sampling), G2 = group 2 (manual sampling) and G3 = group 3 (image derived input function).

When using the arterial input function obtained with the automatic blood sampler (Group 1), both kinetic modelling methods of analysis (2T and Logan with t* = 25 minutes) were found to fit the data (Table 1), although Logan was able to estimate *V*_*T*_ with the lowest %SE (average of 35.7% for 2T and 2.6% for Logan plot) of investigated models (Table 1) albeit with higher Akaike selection criteria (average 99 for 2T versus 131 for Logan plot, S1 Table). The 2T modelling *V*_*T*_ were higher than Logan invasive by ~7–50% (Fig 4). When using the population arterial input function obtained with the manual time-points sampling method (Group 2) and non-invasive image derived input function (Group 3), only Logan was able to fit all regions. The *V*_*T*_ from Group 2 were underestimated by ~48–41% compared with Logan *V*_*T*_ determined using the automatic blood sampling method and the Logan plot (Fig 4). The use of non-invasive image derived input function (Group 3) underestimated Logan *V*_*T*_ by ~88–87% compared with automatic blood sampling input function method (Fig 4). The quantitative underestimation biases were more pronounced in the brain compared with peripheral organs (Fig 4B versus A). Despite measured biases, *V*_*T*_ determined using Logan plot in Group 2 and Group 3 were highly correlated with Group 1 (*r*^*2*^>0.9). As shown in the Bland-Altman plot in Fig 5, there is only one to two outlying measurements for heart (Fig 5A and 5B), cerebellum white matter and frontal association cortex (Fig 5D–5F) across all measurement comparisons. This may be due to poor identifiability of the parameters of the 2T (heart) and Logan (cerebellum white matter and frontal association cortex) models, which results in unreasonably high value for those VOIs *V*_*T*_ and thus may cause deterioration of the overall test-retest metrics. Furthermore, the calculated TEM was lowest for Group 3 (non-invasive image derived input function) independently of inclusion/exclusion of peripheral organs (Fig 6A and 6B, respectively). The %COV was highest for Group 1 (automatic blood sampling) when 2T was used for quantification of *V*_*T*_ and similar across all groups when Logan graphical analysis was used to estimate *V*_*T*_ (Fig 6C and 6D).

**Table 1:** [^18^F]AB5186 kinetic modelling results obtained using 2T compartmental model and Logan graphical analysis with various methods used to derive the input function (mean±SEM, n = 3).

Region/ Tissue	*V*_*T*_− 2T Group 1		*V*_*T*_—Logan Group 1		*V*_*T*_—Logan Group 2		*V*_*T*_—Logan Group 3	
	Mean±S.E.M.	% SE±S.E.M	Mean±S.E.M.	% SE±S.E.M	Mean±S.E.M.	% SE±S.E.M	Mean±S.E.M.	% SE±S.E.M
**Heart**	26.89± 3.17	1.50± 0.24	24.98± 2.85	0.36± 0.07	13.27± 1.11	0.29± 0.09	5.82± 0.20	0.20± 0.07
**Lungs**	7.08± 0.32	16.56± 7.90	6.95± 0.47	0.28± 0.12	3.74± 0.39	0.43± 0.18	1.64± 0.19	0.76± 0.16
**Whole Brain**	3.75± 0.86	15.38± 6.51	3.32± 0.51	2.41± 0.33	1.79± 0.33	2.91± 0.15	0.76± 0.14	3.36± 0.34
**Striatum**	2.53± 0.56	14.87± 4.11	2.39± 0.40	2.09± 0.27	1.28± 0.25	2.57± 0.35	0.55± 0.10	2.65± 0.33
**Frontal Association Cortex**	4.23± 1.46	32.96± 8.10	4.07± 0.86	8.67± 2.03	2.20± 0.54	9.00± 1.56	0.94± 0.22	8.30± 2.20
**Medial Prefrontal Cortex**	3.51± 1.72	223.80± 198.12	2.18± 0.36	2.90± 0.87	1.18± 0.23	3.21± 0.61	0.51± 0.10	3.14± 0.97
**OrbitoFrontal Cortex**	4.24± 0.69	32.22± 9.22	3.59± 0.64	3.57± 0.33	1.94± 0.41	4.04± 0.39	0.83± 0.18	4.04± 0.40
**Enthorhirnal Cortex**	6.16± 1.06	40.40± 8.62	4.13± 0.56	3.91± 0.17	2.23± 0.37	4.54± 0.53	0.93± 0.17	4.81± 0.17
**Hypothalamus**	2.98± 0.38	23.06± 6.94	2.86± 0.33	2.12± 0.15	1.54± 0.23	2.57± 0.53	0.66± 0.10	2.65± 0.18
**Thalamus**	2.44± 0.43	13.72± 2.11	2.49± 0.39	1.47± 0.47	1.34± 0.25	1.83± 0.30	0.58± 0.10	2.10± 0.47
**Midbrain**	2.63± 0.64	27.25± 17.32	2.48± 0.38	1.77± 0.63	1.34± 0.25	2.12± 0.40	0.58± 0.10	2.45± 0.67
**Cerebellum White Matter**	3.35± 0.62	11.45± 5.00	3.25± 0.41	1.79± 0.40	1.75± 0.27	2.23± 0.44	0.75± 0.11	2.50± 0.40
**Cerebellum Grey Matter**	3.94± 0.94	11.22± 3.56	4.20± 0.92	2.79± 0.61	2.03± 0.42	2.95± 0.35	0.86± 0.16	3.34± 0.53

*V*_*T*_, total distribution volume. SEM, standard error of the mean. SE, standard error. Group 1, Swisstrace input function (automatic blood sampling). Group 2, manual input function. Group 3, image derived input function.

## Discussion

This study investigated the quantification bias of kinetic modelling outcome measures of rodent TSPO PET when using image derived input function versus two invasive blood sampling methods. To our knowledge, this is the first study to date investigating such quantification bias of a TSPO PET radiotracer in rodents. Results collected here showed that [^18^F]AB5186 has a consistent distribution with TSPO expression [9,10] and comparable outcome measures to other previously developed radiotracers. For example, injection of [^11^C]PK11195 into naïve Sprague-Dawley rats yielded a *V*_*T*_ in brain of 3.2 mL/cm^3^ [25], versus our [^18^F]AB5186 estimated *V*_*T*_ in brain of 3.3 mL/cm^3^ using the automatic blood sampling invasive quantification methods. Metabolism of [^18^F]AB5186 in rat blood was slower than other previously developed TSPO radiotracers in rats, namely [^18^F]DPA-714 [26], [^18^F]GE180 [27] and [^11^C]DPA-713 [28].

The pharmacokinetics of [^18^F]AB5186 were reversible and could be described by both the 2T model and the invasive Logan graphical analysis when using automatic blood sampling methods (Group 1). The Logan plot was able to fit all regions for Groups 1 to 3 (with lowest %SE), while 2T was only able to fit the data for Group 1, thus and despite higher AIC compared with 2T invasive, the Logan plot was the preferred model to describe [^18^F]AB5186 PET data and allow comparison of study Groups. This is likely due to two main factors: (1) poorly described arterial input function when using population manual sampling approaches, where peak definition is suboptimal; and (2) substantial noise and spill-over issues with image derived input function. Manual sampling methods (Group 2) and image derived input function methods (Group 3) underestimated *V*_*T*_ by approximately 50% and 88% compared with automated sampling, respectively. The underestimation bias of image derived input function observed is likely due to poor blood pool VOI spill over ratios, which deteriorate over time due to radiotracer uptake in surrounding heart tissue. Moreover, sampling requirements for dynamic image-derived input function can impact on accurate description of peak input function (fast perfusion phase) [29] and this is particularly troublesome in small animal PET imaging, owing to the fast heart rate (c. 300–400 bpm) and limited count rate statistics.

Previous studies in mice and rats with [^18^F]FDG have shown that the choice of the blood pool VOI placement (e.g. left ventricle and inferior vena cava) has a profound effect on kinetic outcome measures [30,31]. From those studies, the use of the inferior vena cava was proposed as a reliable and reproducible image derived input function method for [^18^F]FDG kinetic modelling in mice, even without partial-volume corrections. Albeit useful in mice PET studies, as the whole body is in the field of view of preclinical PET systems, the use of the vena cava would be of limited value when collecting dynamic PET data in rats owing to limited size of most scanners field of view and larger animal body size. This is particularly critical when conducting dynamic brain PET studies. The use of the left ventricle blood pool VOI is, therefore, a straightforwardly available option for dynamic PET rat studies of the thorax and brain.

Importantly, Fang and Muzic Jr have shown that, when the blood pool VOI was placed inside the left ventricle (similar to our study with [^18^F]AB5186), the spillover and partial-volume effects of image derived input functions in mice and rats following intravenous injection of [^18^F]FDG could be corrected by a physiological model-corrected input function [32]. This model was able to reduce errors on absolute kinetic modelling outcome measures by at least a 10-fold factor. It is foreseeable that such a method could be equally applied to TSPO PET imaging studies in rodents to reduce errors in the image derived input function method. TSPO is expressed in tissue throughout the body [33], and as a result the use of a reference region such as skeletal muscle is inappropriate. As a result, this study made use of a blood pool VOI.

Conceptually, manual arterial blood sampling methods are considered the gold standard for input function determination, as these unlike original automatic sampling methods are not affected by dead volume issues and background subtraction problems [34,35], nor are these affected by spill-over ratio errors associated with image derived methods. However, in small animals, arterial manual sampling techniques are impractical owing to small blood volume of rodents, and the reduction of blood volume required to derive a good quality input function may influence the animal’s physiology [36]; which consequently could impact on radiotracer uptake kinetics. Therefore, the use of a population average input function for rodent PET data quantification has been suggested as an alternative approach to single animal manual sampling methods [37]. Using [^18^F]FDG in mice, Meyer *et al*. demonstrated that population average input functions can result in large errors in individual animal quantification (30 to 50%), thus introducing variability on PET outcome measures. In this study with [^18^F]AB5186 in rats, we found a bias of approximately 50% when using manual sampling methods (population based) relative to automatic blood sampling methods (individual animal based). This agrees with results from Meyers et al., despite the differences in radiotracer used and animal species, suggesting this might be a fundamental methodological issue rather than radiotracer specific variability.

With the recent development of improved automatic blood samplers [19], the main original issues with this technology (namely, dead volume issues and background subtraction problems) have been resolved. Still, this method requires surgical cannulation and an arterial-venous shunt; deeming this a suboptimal approach for multiple-point longitudinal scanning of small animals. In this study, we have found that, despite measured bias of different input function methods, there was a strong correlation of the kinetic outcome measures determined using image derived methods versus manual sampling versus automatic blood sampling methods. Moreover, on average, the *V*_*T*_ variability per group (assessed by TEM) was lower when using image derived input functions compared with automatic sampling or manual sampling. This is likely due to blood measurement errors leading to an increase of variability when using invasive methods versus in-study/subject image derived input functions. Consequently, the least invasive approach of image-derived input function seems acceptable for quantification of rodent TSPO PET studies. Importantly, the %COV was consistent across all groups when using Logan graphical analysis, thus indicating image derived input function can produce reliable data with similar variation in the outcome measures; albeit biased relative to invasive methods.

## Conclusions

Quantification of TSPO PET rat data using [^18^F]AB5186 can be achieved using image derived input function methods with a VOI placed inside the left ventricle. Despite bias relative to invasive methods of quantification, outcome measures obtained using image derived input functions are robust, strongly agree with outcome measures from invasive methods, are less variable and potentially more amenable for longitudinal scanning of small animals. These results may encourage others to undertake kinetic modelling of rodent PET data due to the suitability and feasibility of image derived input functions.

## Supporting information

S1 Fig Representative radiochromatograms of ^18^F-AB5186 parent peak and radiometabolites. **A)** Parent and radiometabolites of ^18^F-AB5186 at 2 hours post-radiotracer injection in the arterial blood. **B)** Parent peak of ^18^F-AB5186 at 2 hours post-radiotracer injection in the heart and **C)** brain.
(DOCX)

S1 Table Akaike information criterion for each modelling approach and input function method. (DOCX)

S1 Data Synthetic route for AB5186 precursor and standard supplementary file. (DOCX)

S2 Data Raw [^18^F]AB5186 supplementary data file. (XLSX)
