# Supplementary material for: Kinetic modelling and quantification bias in small animal PET studies with [18F]AB5186, a novel 18 kDa translocator protein radiotracer
Source: PLoS One. 2019 May 31;14(5):e0217515. doi: 10.1371/journal.pone.0217515 (PMC6544349; doi:10.1371/journal.pone.0217515)
Supplement: S1 Fig — A) Parent and radiometabolites of 18F-AB5186 at 2 hours post-radiotracer injection in the arterial blood. B) Parent peak of 18F-AB5186 at 2 hours post-radiotracer injection in the heart and C) brain. (DOCX) [file pone.0217515.s001.docx]

**SUPPLEMENTARY FILE**

**Kinetic modelling and quantification bias in small animal PET studies with ^18^F-AB5186, a novel 18 kDa translocator protein radiotracer**

Mark G. MacAskill^1,2^, Tashfeen Walton^1,2^, Lewis Williams^3^, Timaeus E. F. Morgan^3^, Carlos José Alcaide-Corral^1,2^, Marc R. Dweck^1^, Gillian A. Gray^1^, David E. Newby^1^, Christophe Lucatelli^2^, Andrew Sutherland^3^, Sally L. Pimlott^4,5^, Adriana A.S. Tavares^1,2,*^

^1^ University/ BHF Centre for Cardiovascular Science, University of Edinburgh, Edinburgh, UK.

^2^ Edinburgh Imaging, University of Edinburgh, Edinburgh, UK.

^3^ WestCHEM, School of Chemistry, University of Glasgow, UK.

^4^ School of Medicine, University of Glasgow, UK.

^5^ West of Scotland PET Centre, NHS Greater Glasgow and Clyde, UK.


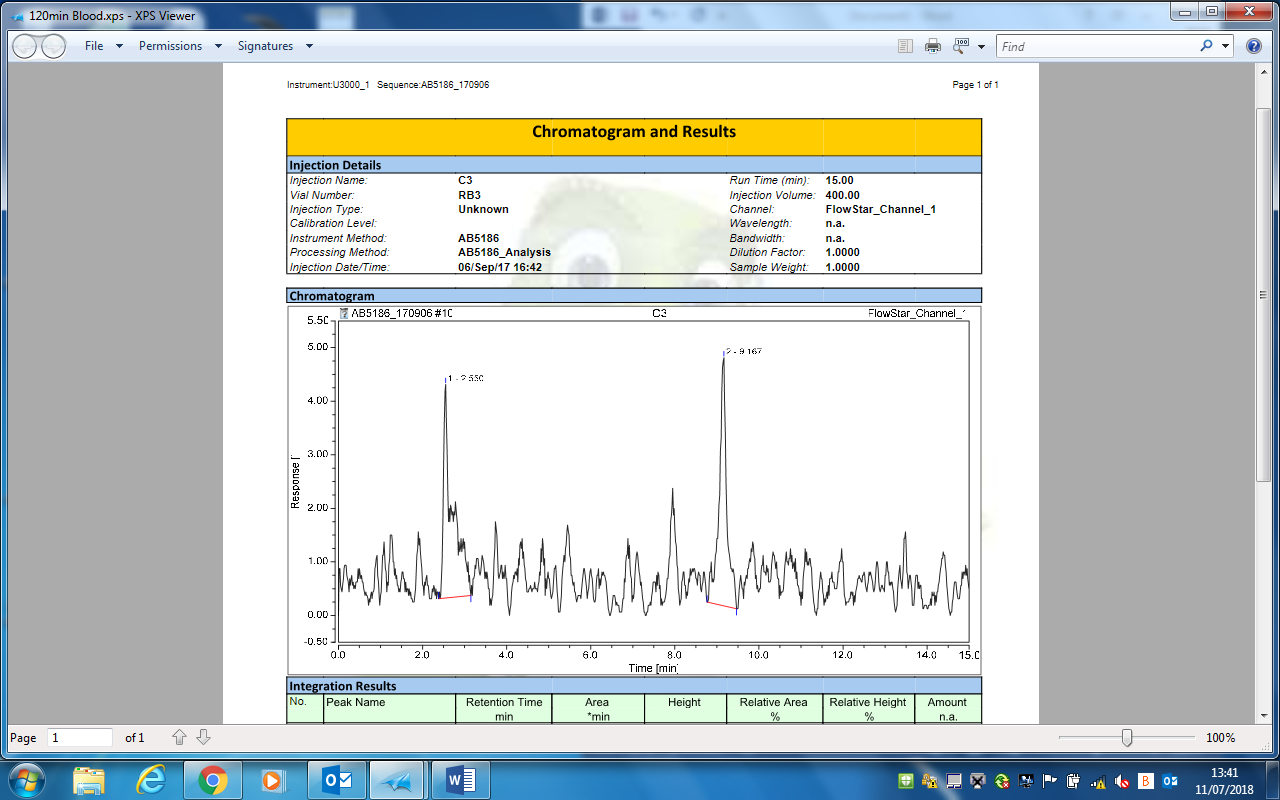

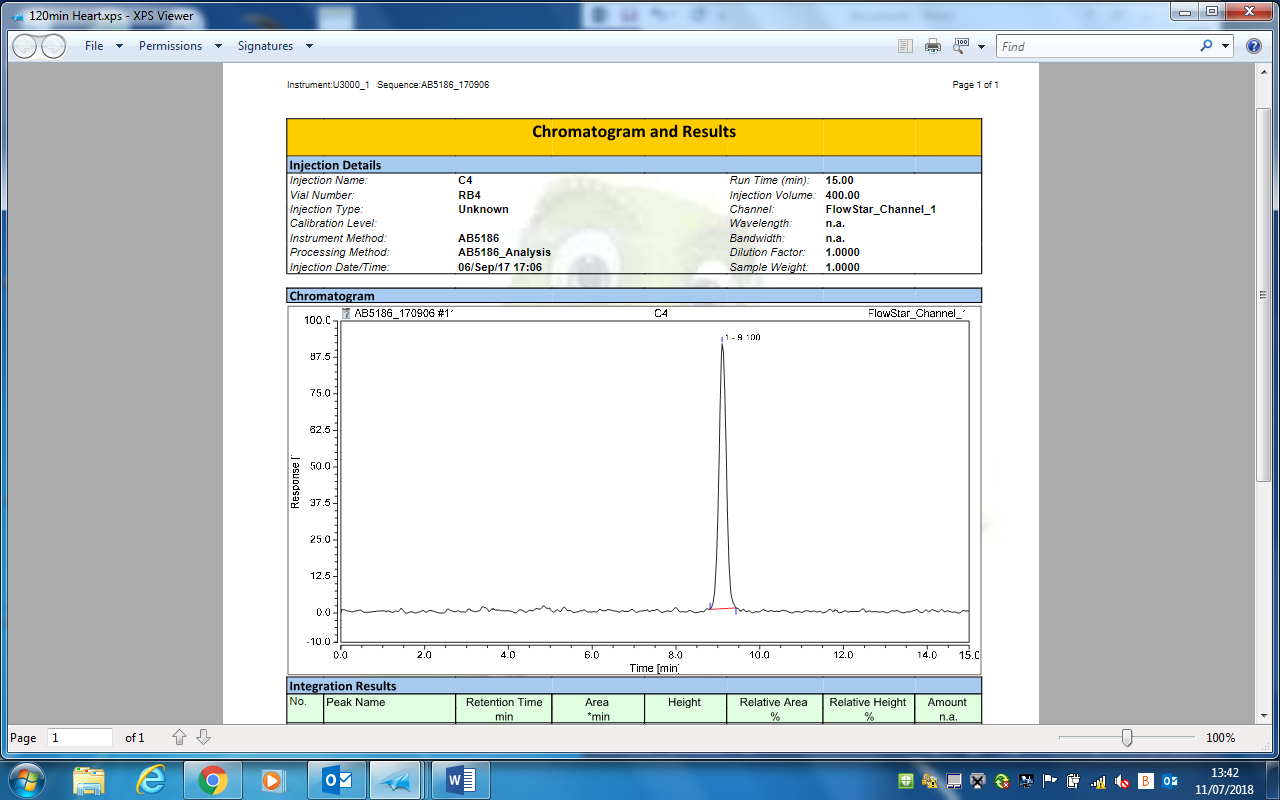

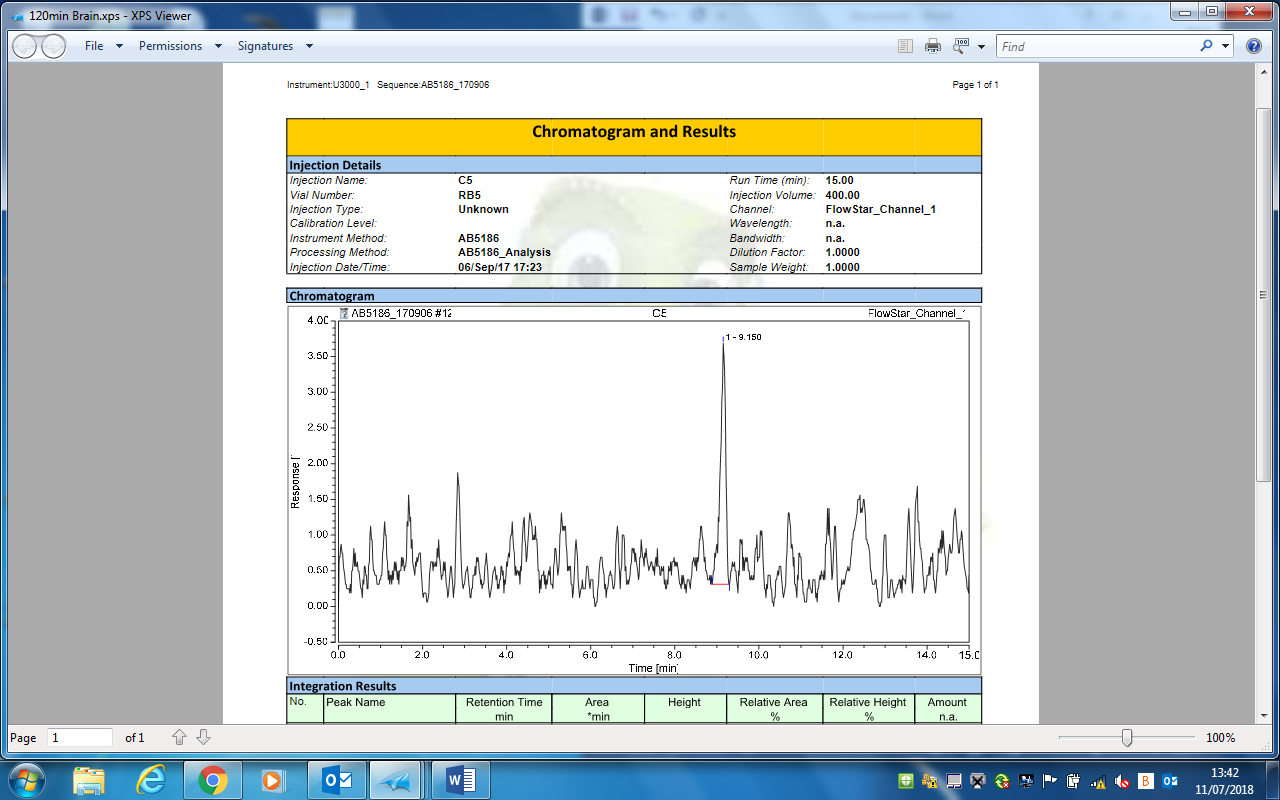


**A**

**B**

**C**

**S1 Fig. Representative radiochromatograms of ^18^F-AB5186 parent peak and radiometabolites.** **A)** Parent and radiometabolites of ^18^F-AB5186 at 2 hours post-radiotracer injection in the arterial blood. **B)** Parent peak of ^18^F-AB5186 at 2 hours post-radiotracer injection in the heart and **C)** brain.
