# Supplementary material for: Kinetic modelling and quantification bias in small animal PET studies with [18F]AB5186, a novel 18 kDa translocator protein radiotracer
Source: PLoS One. 2019 May 31;14(5):e0217515. doi: 10.1371/journal.pone.0217515 (PMC6544349; doi:10.1371/journal.pone.0217515)
Supplement: S1 Table — (DOCX) [file pone.0217515.s002.docx]

**SUPPLEMENTARY FILE**

**Kinetic modelling and quantification bias in small animal PET studies with ^18^F-AB5186, a novel 18 kDa translocator protein radiotracer**

Mark G. MacAskill^1,2^, Tashfeen Walton^1,2^, Lewis Williams^3^, Timaeus E. F. Morgan^3^, Carlos José Alcaide-Corral^1,2^, Marc R. Dweck^1^, Gillian A. Gray^1^, David E. Newby^1^, Christophe Lucatelli^2^, Andrew Sutherland^3^, Sally L. Pimlott^4,5^, Adriana A.S. Tavares^1,2,*^

^1^ University/ BHF Centre for Cardiovascular Science, University of Edinburgh, Edinburgh, UK.

^2^ Edinburgh Imaging, University of Edinburgh, Edinburgh, UK.

^3^ WestCHEM, School of Chemistry, University of Glasgow, UK.

^4^ School of Medicine, University of Glasgow, UK.

^5^ West of Scotland PET Centre, NHS Greater Glasgow and Clyde, UK.

| **Region/ Tissue** | **AIC – 2T Group 1** | **AIC - Logan Group 1** | **AIC - Logan Group 2** | **AIC - Logan Group 3** |
| --- | --- | --- | --- | --- |
|  | **Mean± S.E.M.** | **Mean± S.E.M.** | **Mean± S.E.M.** | **Mean± S.E.M.** |
| **Heart** | 19.49± 8.44 | 102.09± 5.07 | 94.08± 9.35 | 83.45± 9.81 |
| **Lungs** | 102.79± 17.25 | 86.67± 13.97 | 95.12± 17.97 | 117.34± 4.53 |
| **Whole Brain** | 67.86± 15.98 | 134.89± 3.18 | 140.23± 1.86 | 143.67± 2.10 |
| **Striatum** | 89.50± 7.82 | 133.84± 2.04 | 139.15± 3.89 | 140.05± 1.93 |
| **Frontal Association Cortex** | 156.84± 12.79 | 163.81± 4.68 | 165.67± 2.52 | 162.12± 5.97 |
| **Medial Prefrontal Cortex** | 160.37± 7.87 | 141.55± 6.53 | 145.88± 2.71 | 143.37± 6.76 |
| **OrbitoFrontal Cortex** | 110.83± 12.91 | 145.21± 1.47 | 148.29± 1.87 | 148.33± 1.84 |
| **Enthorhirnal Cortex** | 73.48± 10.97 | 144.62± 1.31 | 148.14± 3.32 | 149.91± 0.85 |
| **Hypothalamus** | 116.48± 10.82 | 134.15± 1.82 | 138.17± 4.79 | 139.95± 1.30 |
| **Thalamus** | 104.91± 5.66 | 125.40± 7.74 | 133.10± 3.80 | 135.99± 4.93 |
| **Midbrain** | 111.73± 13.37 | 127.66± 9.40 | 135.68± 2.87 | 138.17± 5.74 |
| **Cerebellum White Matter** | 81.03± 23.44 | 130.12± 4.55 | 135.68± 5.00 | 139.28± 3.15 |
| **Cerebellum Grey Matter** | 85.51± 4.35 | 135.15± 4.12 | 140.47± 2.75 | 143.47± 3.04 |

**S1 Table. Akaike information criterion for each modelling approach and input function method.**
