# Supplementary material for: Kinetic modelling and quantification bias in small animal PET studies with [18F]AB5186, a novel 18 kDa translocator protein radiotracer
Source: PLoS One. 2019 May 31;14(5):e0217515. doi: 10.1371/journal.pone.0217515 (PMC6544349; doi:10.1371/journal.pone.0217515)
Supplement: S1 Data — (DOCX) [file pone.0217515.s003.docx]

**SUPPLEMENTARY FILE**

**Kinetic modelling and quantification bias in small animal PET studies with ^18^F-AB5186, a novel 18 kDa translocator protein radiotracer**

Mark G. MacAskill^1,2^, Tashfeen Walton^1,2^, Lewis Williams^3^, Timaeus E. F. Morgan^3^, Carlos José Alcaide-Corral^1,2^, Marc R. Dweck^1^, Gillian A. Gray^1^, David E. Newby^1^, Christophe Lucatelli^2^, Andrew Sutherland^3^, Sally L. Pimlott^4,5^, Adriana A.S. Tavares^1,2,*^

^1^ University/ BHF Centre for Cardiovascular Science, University of Edinburgh, Edinburgh, UK.

^2^ Edinburgh Imaging, University of Edinburgh, Edinburgh, UK.

^3^ WestCHEM, School of Chemistry, University of Glasgow, UK.

^4^ School of Medicine, University of Glasgow, UK.

^5^ West of Scotland PET Centre, NHS Greater Glasgow and Clyde, UK.

**S1 Data:**

**Synthetic Route For AB5186 Precursor And Standard**

**General Experimental**

All reagents and starting materials were obtained from commercial sources and used as received. All dry solvents were purified using a PureSolv 500 MD solvent purification system. All reactions were performed under argon unless otherwise stated. Brine is defined as a saturated solution of aqueous sodium chloride. Flash column chromatography was carried out using Fisher Matrix silica 60. Macherey–Nagel aluminium–backed plates pre–coated with silica gel 60 (UV254) were used for thin layer chromatography and were visualized using UV light. ^1^H NMR and ^13^C NMR spectra were recorded on a Bruker DPX 400 spectrometer or Bruker 500 spectrometer with chemical shift values in ppm relative to tetramethylsilane (δ_H_ 0.00 and δ_C_ 0.0) or residual chloroform (δ_H_ 7.26 and δ_C_ 77.2) as the standard. ^1^H and ^13^C assignments are based on two-dimensional COSY and DEPT experiments, respectively. Infrared spectra were recorded on a JASCO FTIR 410 spectrometer. Mass spectra were recorded using electron impact, chemical ionization or fast atom bombardment techniques. HRMS spectra were recorded using a dual-focusing magnetic analyzer mass spectrometer. Melting points were determined on a Gallenkamp melting point apparatus.

**Experimental Procedures And Spectroscopic Data**

**Diethyl 4-phenylquinoline-2,3-dicarboxylate^1^**

Indium(III) chloride (0.680 g, 3.07 mmol) was added to 2-aminobenzophenone (5.00 g, 15.3 mmol) and diethyl acetylenedicarboxylate (2.95 mL, 18.4 mmol), and the neat mixture stirred at 80 °C for 3 h. On cooling to ambient temperature, water (50 mL) was added and the mixture extracted using ethyl acetate (150 mL). The organic layer was then washed with water (100 mL) and brine (100 mL), dried (MgSO_4_), filtered and concentrated *in vacuo*. Purification by silica column chromatography (ethyl acetate/hexane, 15:85) afforded diethyl 4-phenylquinoline-2,3-dicarboxylate (5.36 g, 100%) as a yellow solid. Mp 89–91 °C (lit.^1^ mp 95–96 °C); IR (neat) 2984 (CH), 1741 (CO), 1722 (CO), 1560 (C=C), 1441, 1376, 1247, 1202, 1048, 768 cm^–1^; ^1^H NMR (400 MHz, CDCl_3_) δ 0.99 (3H, t, *J* = 7.2 Hz, OCH_2_C*H*_3_), 1.47 (3H, t, *J* = 7.2 Hz, OCH_2_C*H*_3_), 4.09 (2H, q, *J* = 7.2 Hz, OC*H*_2_CH_3_), 4.54 (2H, q, *J* = 7.2 Hz, OC*H*_2_CH_3_), 7.34–7.40 (2H, m, 2 × ArH), 7.47–7.53 (3H, m, 3 × ArH), 7.57 (1H, ddd, *J* = 8.4, 6.4, 1.6 Hz, ArH), 7.63 (1H, ddd, *J* = 8.4, 1.6, 0.6 Hz, ArH), 7.81 (1H, ddd, *J* = 8.4, 6.4, 1.6 Hz, ArH), 8.38 (1H, ddd, *J* = 8.4, 1.6, 0.6 Hz, ArH); ^13^C NMR (101 MHz, CDCl_3_) δ 13.6 (CH_3_), 14.2 (CH_3_), 61.6 (CH_2_), 62.6 (CH_2_), 126.6 (CH), 127.1 (C), 127.5 (C), 128.2 (2 × CH), 128.7 (CH), 129.0 (CH), 129.4 (2 × CH), 130.7 (CH), 130.9 (CH), 134.8 (C), 145.9 (C), 147.1 (C), 148.0 (C), 165.3 (C), 167.2 (C); MS (EI) *m/z* 349 (M^+^, 16%), 305 (20), 276 (55), 205 (100), 204 (82), 165 (12), 84 (5); HRMS (EI) calcd for C_21_H_19_NO_4_ (M^+^), 349.1314, found 349.1311.

**2,3-Bis(hydroxymethyl)-4-phenylquinoline^2^**

A suspension of lithium aluminium hydride (3.90 g, 103 mmol) in diethyl ether (90 mL) was cooled to 0 °C and this was added dropwise to a solution of diethyl 4-phenylquinoline-2,3-dicarboxylate (9.00 g, 26.0 mmol) in diethyl ether (90 mL). The temperature of the reaction mixture was maintained at 0 °C and stirred vigorously for 3 h. After gradually warming to ambient temperature, the mixture was quenched by the dropwise addition of a 10% aqueous solution of potassium sodium tartrate (100 mL) and stirred for 3 h. The resultant mixture was extracted with diethyl ether (3 × 100 mL) and the combined organic extracts were dried (Na_2_SO_4_), filtered and concentrated *in vacuo* to afford a colourless residue that was dissolved in methanol (250 mL). 10% Palladium on carbon (0.90 g) was added to the reaction flask and the resultant suspension stirred at ambient temperature for 15 h. The suspension was then filtered through a pad of Celite^®^, washed with methanol and concentrated *in vacuo*. Trituration with diethyl ether afforded 2,3-bis(hydroxymethyl)-4-phenylquinoline as a white solid (5.33 g, 78%). Mp 171–173 °C (lit.^2^ mp 175–177 °C); IR (neat) 3422 (OH), 3074, 2991 (CH), 1571, 1490, 1442, 1231, 1022, 1006 cm^–1^; ^1^H NMR (400 MHz, CD_3_OD) δ 4.59 (2H, s, CH_2_OH), 5.11 (2H, s, CH_2_OH), 7.34–7.38 (2H, m, 2 × ArH), 7.41 (1H, dd, *J* = 8.4, 1.2 Hz, ArH), 7.44–7.50 (1H, m, ArH), 7.51–7.60 (3H, m, 3 × ArH), 7.73 (1H, ddd, *J* = 8.4, 6.4, 1.2 Hz, ArH), 8.11 (1H, d, *J* = 8.4 Hz, ArH); ^13^C NMR (101 MHz, CD_3_OD) δ 59.1 (CH_2_), 64.6 (CH_2_), 127.8 (CH), 127.9 (CH), 128.6 (C), 129.4 (CH), 129.5 (CH), 129.5 (2 × CH), 130.0 (C), 130.8 (CH), 130.8 (2 × CH), 137.2 (C), 147.4 (C), 150.4 (C), 160.9 (C); MS (CI) *m/z* 266 (M+H^+^, 100%), 264 (50), 248 (42), 218 (21), 206 (4), 151 (3); HRMS (CI) calcd for C_17_H_16_NO_2_ (M+H^+^), 266.1181, found 266.1186.

**9-Phenylfuro[3,4-*b*]quinolin-3(*1H*)-one^2^**

To a solution of 2,3-bis(hydroxymethyl)-4-phenylquinoline (0.780 g, 2.93 mmol) in chloroform (50 mL) was added activated manganese(IV) oxide (7.64 g, 87.9 mmol) in one portion. The resultant suspension was stirred vigorously at ambient temperature for 4 h and then filtered through Celite^®^. After washing with chloroform (100 mL), the solvent was removed *in vacuo* to afford 9-phenylfuro[3,4-*b*]quinolin-3(*1H*)-one as a white solid (0.70 g, 92%), which was used without further purification. Mp 188–190 °C (lit.^2^ mp 190–192 °C); IR (neat) 3005 (CH), 1775 (CO), 1582, 1414, 1371, 1152, 1121, 1055, 1007 cm^–1^; ^1^H NMR (400 MHz, CDCl_3_) δ 5.41 (2H, s, OCH_2_), 7.45 (2H, dd, *J* = 8.0, 2.0 Hz, 2 × ArH), 7.57–7.70 (4H, m, 4 × ArH), 7.84–7.90 (1H, m, ArH), 7.92 (1H, d, *J* = 8.4 Hz, ArH), 8.46 (1H, d, *J* = 8.4 Hz, ArH); ^13^C NMR (101 MHz, CDCl_3_) δ 67.8 (CH_2_), 125.7 (CH), 127.9 (C), 128.9 (2 × CH), 129.3 (2 × CH), 129.4 (CH), 129.6 (CH), 130.7 (CH), 131.5 (CH), 132.3 (C), 133.6 (C), 143.9 (C), 144.4 (C), 150.7 (C), 168.7 (C); MS (ESI) *m/z* 284 (M+Na^+^, 100%); HRMS (ESI) calcd for C_17_H_11_NNaO_2_ (M+Na^+^), 284.0682, found 284.0677.

**3-(Hydroxymethyl)-4-phenylquinoline-2-*N*-diethylcarboxamide^3^**

To a suspension of aluminium(III) chloride (0.76 g, 5.7 mmol) in dichloromethane (25 mL) was added dropwise, triethylamine (0.60 mL, 5.7 mmol). The resultant solution was stirred for 0.5 h, before addition of a solution of 9-phenylfuro[3,4-*b*]quinolin-3(*1H*)-one (0.50 g, 1.9 mmol) in dichloromethane (25 mL). The resultant solution was stirred for 15 h at room temperature, before quenching by addition of an aqueous solution of 2 M hydrochloric acid (25 mL). The mixture was extracted with dichloromethane (3 × 20 mL). The combined organic extracts were dried (Na_2_SO_4_), filtered and concentrated *in vacuo*. The crude product was purified by column chromatography (petroleum ether/ethyl acetate, 3:1) to yield the title compound as a white solid (0.61 g, 95%). Spectroscopic data was consistent with the literature.^3^ Mp 117–119 °C; IR (neat) 3408 (OH), 2972, 2932 (CH), 1613 (CO), 1483, 1443, 1267, 1216, 1069 cm^–1^; ^1^H NMR (400 MHz, CDCl_3_) δ 1.27 (3H, t, *J* = 7.2 Hz, NCH_2_C*H*_3_), 1.36 (3H, t, *J* = 7.2 Hz, NCH_2_C*H*_3_), 3.42 (2H, q, *J* = 7.2 Hz, NC*H*_2_CH_3_), 3.68 (2H, q, *J* = 7.2 Hz, NC*H*_2_CH_3_), 4.12 (1H, t, *J* = 6.4 Hz, CH_2_O*H*), 4.39 (2H, d, *J* = 6.4 Hz, C*H*_2_OH), 7.41–7.58 (7H, m, 7 × ArH), 7.72 (1H, ddd, *J* = 8.4, 6.8, 1.6 Hz, ArH), 8.12 (1H, ddd, *J* = 8.4, 1.2, 0.8 Hz, ArH); ^13^C NMR (101 MHz, CDCl_3_) δ 12.9 (CH_3_), 14.2 (CH_3_), 40.3 (CH_2_), 43.7 (CH_2_), 59.7 (CH_2_), 127.0 (CH), 127.4 (CH), 127.6 (C), 128.4 (3 × CH), 129.4 (C), 129.6 (CH), 129.7 (CH), 129.9 (2 × CH), 135.2 (C), 146.1 (C), 149.3 (C), 155.1 (C), 169.8 (C); MS (ESI) *m/z* 357 (M+Na^+^, 100%); HRMS (ESI) calcd for C_21_H_22_N_2_NaO_2_ (M+Na^+^), 357.1573, found 357.1576.

**3-(Chloromethyl)-4-phenylquinoline-2-*N*-diethylcarboxamide^3^**

To a solution of 3-(hydroxymethyl)-4-phenylquinoline-2-*N*-diethylcarboxamide (0.10 g, 0.30 mmol) in dichloromethane (10 mL) was added thionyl chloride (0.65 mL, 9.0 mmol) and the reaction mixture stirred under reflux for 24 h. On cooling to ambient temperature, the mixture was concentrated *in vacuo* and azeotroped with toluene (3 × 10 mL) to remove excess thionyl chloride. Purification by silica column chromatography (diethyl ether/petroleum ether, 1:1) gave 3-(chloromethyl)-4-phenylquinoline-2-*N*-diethylcarboxamide as a clear oil (0.11 g, 100%). Spectroscopic data was consistent with the literature.^3^ IR (neat) 2981 (CH), 2936, 1630 (CO), 1560 (C=C), 1482, 1443, 1270, 1218, 1127, 1067, 728 cm^–1^; ^1^H NMR (500 MHz, CDCl_3_) δ 1.30 (3H, t, *J* = 7.0 Hz, NCH_2_C*H*_3_), 1.35 (3H, t, *J* = 7.5 Hz, NCH_2_C*H*_3_), 3.28 (2H, q, *J* = 7.0 Hz, NC*H*_2_CH_3_), 3.68 (2H, q, *J* = 7.5 Hz, NC*H*_2_CH_3_), 4.70 (2H, s, CH*_2_*Cl), 7.36–7.40 (2H, m, 2 × ArH), 7.41–7.49 (2H, m, 2 × ArH), 7.51–7.59 (3H, m, 3 × ArH), 7.73 (1H, ddd, *J* = 8.5, 6.5, 1.5 Hz, ArH), 8.13 (1H, d, *J* = 8.5 Hz, ArH); ^13^C NMR (126 MHz, CDCl_3_) δ 12.3 (CH_3_), 13.5 (CH_3_), 39.2 (CH_2_), 40.3 (CH_2_), 43.6 (CH_2_), 125.9 (C), 126.9 (CH), 127.3 (C), 127.4 (CH), 128.6 (2 × CH), 128.7 (CH), 129.4 (2 × CH), 129.6 (CH), 130.2 (CH), 134.9 (C), 146.4 (C), 149.4 (C), 155.6 (C), 168.1 (C); MS (ESI) *m/z* 375 (M+Na^+^, 100%); HRMS (ESI) calcd for C_21_H_21_^35^ClN_2_NaO (M+Na^+^), 375.1235, found 375.1218.

**3-(Fluoromethyl)-4-phenylquinoline-2-*N*-diethylcarboxamide^3^**

To a solution of 18-crown-6 (0.040 g, 0.14 mmol) in acetonitrile (2.5 mL) was added potassium fluoride (0.040 g, 0.71 mmol) and the resultant suspension stirred at ambient temperature for 0.5 h. A solution of 3-(chloromethyl)-4-phenylquinoline-2-*N*-diethylcarboxamide (0.050 g, 0.14 mmol) in acetonitrile (2.5 mL) was then added dropwise to the reaction mixture and stirred under reflux for 24 h. On cooling to ambient temperature, water (10 mL) was added to the mixture, which was then extracted using dichloromethane (3 × 15 mL). The organic layer was dried (MgSO_4_), filtered and concentrated *in vacuo*. Purification by silica column chromatography (diethyl ether/petroleum ether, 1:1), followed by washing with acetonitrile gave 3-(fluoromethyl)-4-phenylquinoline-2-*N*-diethylcarboxamide as a white solid (0.030 g, 60%). Spectroscopic data was consistent with the literature.^3^ IR (neat) 2976, 2936 (CH), 1634 (CO), 1560 (C=C), 1481, 1269, 1213, 1069, 970 cm^–1^; ^1^H NMR (400 MHz, CDCl_3_) δ 1.22 (3H, t, *J* = 7.2 Hz, NCH_2_C*H*_3_), 1.34 (3H, t, *J* = 7.2 Hz, NCH_2_C*H*_3_), 3.28 (2H, q, *J* = 7.2 Hz, NC*H*_2_CH_3_), 3.67 (2H, q, *J* = 7.2 Hz, NC*H*_2_CH_3_), 5.38 (2H, d, *J* = 47.8 Hz, CH_2_F), 7.32–7.38 (2H, m, 2 × ArH), 7.46–7.57 (5H, m, 5 × ArH), 7.73–7.78 (1H, m, ArH), 8.16 (1H, d, *J* = 8.4 Hz, ArH); ^13^C NMR (101 MHz, CDCl_3_) δ 12.6 (CH_3_), 13.7 (CH_3_), 39.4 (CH_2_), 43.2 (CH_2_), 79.2 (CH_2_, d, ^1^*J*_C-F_ = 162.2), 123.4 (C, d, ^2^*J*_C-F_ = 15.1 Hz), 127.0 (CH), 127.1 (C, d, ^4^*J*_C-F_ = 1.8 Hz), 127.4 (CH), 128.5 (2 × CH), 128.7 (CH), 129.6 (CH), 129.7 (2 × CH), 130.5 (CH, d, ^5^*J*_C-F_ = 1.5 Hz), 134.8 (C), 147.1 (C, d, ^3^*J*_C-F_ = 2.9 Hz), 150.7 (C, d, ^3^*J*_C-F_ = 4.9 Hz), 155.9 (C, d, ^4^*J*_C-F_ = 1.7 Hz), 168.2 (C); MS (ESI) *m/z* 359 (M+Na^+^, 100%); HRMS (ESI) calcd for C_21_H_21_FN_2_NaO (M+Na^+^), 359.1530, found 359.1520.

**References**

1. Chanda T, Verma RK, Singh MS. InCl_3_-Driven Regioselective Synthesis of Functionalized/Annulated Quinolines: Scope and Limitations. Chem Asian J. 2012;7:778–787.

2. Anzini M, Cappelli A, Vomero, S. Synthesis of 2-Substituted 2,3-Dihydro-9-phenyl-1H-Pyrrolo[3,4-b]quinolin-3-ones as Potential Peripheral Benzodiazepine-Receptor Ligands. Heterocycles. 1994;38:103–111.

3. Blair A, Zmuda, F, Malviya G, Tavares AAS, Tamagnan, GD, Chalmers AJ, Dewar D, Pimlott SL, Sutherland A. A Novel ^18^F-Labelled High Affinity Agent for PET Imaging of the Translocator Protein. Chem Sci. 2015;6:4772–4777.

**NMR Spectra For All Compounds**
